# Glial Cells Are Involved in ANG-II-Induced Vasopressin Release and Sodium Intake in Awake Rats

**DOI:** 10.3389/fphys.2018.00430

**Published:** 2018-05-01

**Authors:** Atalia F. L. Flôr, José L. de Brito Alves, Maria S. França-Silva, Camille M. Balarini, Lucila L. K. Elias, Silvia G. Ruginsk, José Antunes-Rodrigues, Valdir A. Braga, Josiane C. Cruz

**Affiliations:** ^1^Departamento de Biotecnologia, Centro de Biotecnologia, Universidade Federal da Paraíba, João Pessoa, Brazil; ^2^Departamento de Fisiologia e Patologia, Centro de Ciências da Saúde, Universidade Federal da Paraíba, João Pessoa, Brazil; ^3^Departamento de Fisiologia, Faculdade de Medicina de Ribeirão Preto, Universidade de São Paulo, Ribeirão Preto, Brazil; ^4^Departamento de Ciências Fisiológicas, Instituto de Ciências Biomédicas, Universidade Federal de Alfenas, Alfenas, Brazil

**Keywords:** central ANG-II, glial cells, pressor response, neuroendocrine response, drinking behavior

## Abstract

It is known that circulating angiotensin II (ANG-II) acts on the circumventricular organs (CVOs), which partially lack a normal blood-brain barrier, to stimulate pressor responses, vasopressin (AVP), and oxytocin (OT) secretion, as well as sodium and water intake. Although ANG-II type 1 receptors (AT1_R_) are expressed in neurons and astrocytes, the involvement of CVOs glial cells in the neuroendocrine, cardiovascular and behavioral responses induced by central ANG II remains to be further elucidated. To address this question, we performed a set of experiments combining *in vitro* studies in primary hypothalamic astrocyte cells (HACc) and *in vivo* intracerebroventricular (icv) microinjections into the lateral ventricle of awake rats. Our results showed that ANG-II decreased glutamate uptake in HACc. In addition, *in vivo* studies showed that fluorocitrate (FCt), a reversible glial inhibitor, increased OT secretion and mean arterial pressure (MAP) and decreased breathing at rest. Furthermore, previous FCt decreased AVP secretion and sodium intake induced by central ANG-II. Together, our findings support that CVOs glial cells are important in mediating neuroendocrine and cardiorespiratory functions, as well as central ANG-II-induced AVP release and salt-intake behavior in awake rats. In the light of our *in vitro* studies, we propose that these mechanisms are, at least in part, by ANG-II-induced astrocyte mediate reduction in glutamate extracellular clearance.

## Introduction

Hypovolemia produced by dehydration, salt loading or water deprivation induces a marked increase in circulating ANG-II, which modulates body fluid and cardiorespiratory homeostasis through circumventricular organs (CVOs). CVOs are rich in fenestrated capillaries and partially lack the blood brain barrier (Fitzsimons, 1980; Gross et al., 1987; Beresford and Fitzsimons, 1992). Intracerebroventricular microinjections of ANG-II induce: (i) AVP and OT release (Lang et al., 1981; Beresford and Fitzsimons, 1992; Reis et al., 2007, 2010; Sakai et al., 2007), (ii) water and sodium intake (Fitzsimons, 1980; Beresford and Fitzsimons, 1992; Sakai et al., 2007; Reis et al., 2010; Matsuda et al., 2016; Roncari et al., 2017), and (iii) cardiorespiratory responses (Potter and McCloskey, 1979; Alexander and Lumbers, 1981; Bains et al., 1992; Ohtake and Jennings, 1993; Saad et al., 2004; Reis et al., 2010). There is a high density of angiotensinergic receptors (AT1_R_ and AT2_R_) in neurons but also in CVOs astrocytes, mainly from subfornical organ (SFO) and organum vasculosum of the lamina terminalis (OVLT; Gebke et al., 1998). *In vitro* studies have shown that ANG-II in isolated SFO, OVLT neurons or astrocytes induce calcium transients waves (Li and Ferguson, 1993; Gebke et al., 1998), suggesting that these cells could be important intermediates involved in the hydromineral and cardiorespiratory homeostasis induced by central ANG-II. It is known that circulating ANG-II induces new ANG-II synthesis in SFO neurons projecting to pre-autonomic and neuroendocrine portions of the paraventricular nuclei (PVN; Bains et al., 1992; Li and Ferguson, 1993; Sakai et al., 2007; Burmeister et al., 2011; Stern et al., 2016). The PVN has been described as a key angiotensin-sensitive hypothalamic nucleus; integrating neuroendocrine, behavioral and cardiorespiratory responses to hydromineral imbalance (Ferguson, 1988; Bains et al., 1992; Yeh et al., 1997; Schlenker et al., 2001; Stern et al., 2016). Hypothalamic astrocytes appear to be functionally critical for ANG II-induced fluid balance and hemodynamic homeostasis, since an elegant study by Stern et al. (2016), combining *in vitro* patch-clamp and *in vivo* approaches showed that ANG-II in the PVN inhibited astrocyte-specific glutamate transporter (GLT-1) activity, inducing increased extracellular glutamate levels, which contributed to increased pre-sympathetic neuronal activity, sympathoexcitatory outflow, and blood pressure. We hypothesize that neuroendocrine, behavioral and cardiorespiratory responses, induced by central ANG-II, could be mediated by CVOs glial cells. To address this issue, we performed *in vitro* experiments to evaluate ANG-II effects in HACc and *in vivo* experiments analyzing the effects of glial inhibition on the ANG-II-induced neuroendocrine, behavioral, and cardiovascular responses in awake rats.

**Graphical Abstract:**
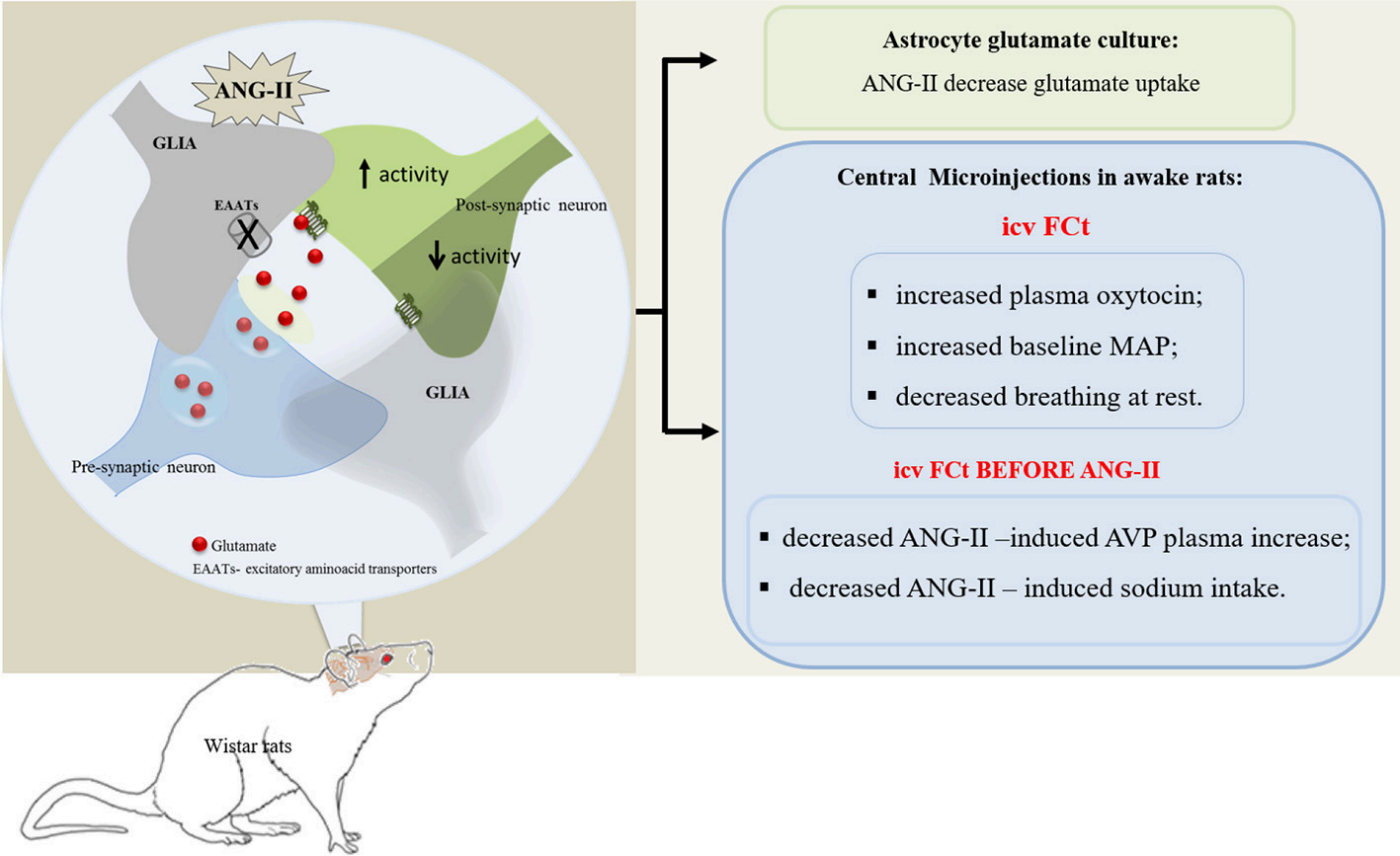
*In vitro* studies showed that ANG-II decreased astrocyte glutamate uptake, in turn, contributing to increase extracellular glutamate bioavailability. In addition, *in vivo* studies showed that CVOs glial cells are important to modulate neuroendocrine and cardiorespiratory homeostasis, including ANG-II-induced AVP release and sodium intake in awake rats.

## Materials and methods

### Ethical approval

All experimental procedures used in this study were approved by Ethics Committee on the Use of Animals of the Biotechnology Center of the Federal University of Paraiba [CEUA-CBIOTEC/UFPB (ref. numbers CEUA no° 0606/13 and CEUA no° 023/15)] or by the Ethics Committee on Animal Use of the School of Medicine of Ribeirao Preto of the University of Sao Paulo [CEUA-FMRP/USP (ref. number n° 95/2011)] and performed according to the guidelines of the Brazilian code of practice for care and use of animals for scientific purposes (Brazilian College of Animal Experimentation-COBEA).

Primary hypothalamic astrocyte cultures were generated from newborn (2–3 days old) male Wistar rats (*Rattus norvegicus*), obtained from the Animal Facility of the School of Medicine of Ribeirao Preto—University of Sao Paulo (FMRP-USP). The rats pups were swiftly decapitated, the brains were dissected and placed in an isotonic salt solution. The medial basal hypothalamus was rostrally delimited by the optic chiasm and mammillary bodies, laterally by the tuber cinereum and by the top of third ventricle, dissected and isolated from the brain. Connective tissues were chemically digested with trypsin (0.05%) and mechanically dissociated (fire-polished glass Pasteur pipette). Cells were grown 7–10 days in a CO_2_ incubator at 37°C in Dulbecco's modified Eagle's medium (DMEM)/F12 (Sigma) containing 10% bovine fetal serum (LGC Biotechnology) and 1% penicillin-streptomycin (Mediatech). Upon reaching confluence, cell cultures were shaken at 200 rpm for 30 min, according to McCarthy and De Vellis (1980). Afterwards, the astrocyte cultures were transferred to 12- or 6-well sterile culture plates (TPP®) with fresh medium.

### Tritiated [^3^H] aspartate uptake assay

[^3^H]aspartate uptake modulated by ANG II was evaluated in HACs. Considering that excitatory amino acid transporter (EAAT) mediate aspartic acid and glutamic transport (Schousboe et al., 2004; Kavanaugh, 2009). Here, [^3^H] aspartate was used as tracer of glutamate to measure GLAST and GLT-1 (EEATs) activity in HACs. Thus, assays were performed with (^3^H)-aspartate [0.1 mCi/mL (PerkinElmer Life and Analytical Sciences)] to evaluate aspartate astrocyte uptake. On the day of the experiment, culture medium was removed and incubated at 37°C for a variable period of time (according to the experimental protocol) with (1) isotonic Hank solution (~290 mOsm/kg H_2_O, 128 mM NaCl, pH 7.4) + 10 μL (0.1 μCi) of tritiated (^3^H)-, assigned as control group; (2) isotonic Hank solution containing ANG II (1, 10, or 100 nM, Peninsula Laboratories) + 10 μL (0.1 μCi) of tritiated (^3^H)-aspartate. For each ANG II concentration we performed a different set of experiments, stimulating HACCs during 5, 10, 15, or 30 min. Thus, each amount of [^3^H]-aspartate taken up by astrocytes was quantified by liquid scintillation counting (Cristovao-Ferreira et al., 2011; Pérez-Domínguez et al., 2014). Afterwards, Hank solution was removed and adhered cells were lysed with cold deionized sterile water (for 2 min) and, with the aid of a cell scraper, were collected into vials containing scintillation liquid (Scintisafetm econo 1, Fisher Scientific sx20-5). Due to the adsorption of plastic surfaces shown by aspartate, a blank well (without cells) was utilized as a measure of background signal. Samples of medium and astrocyte lysates were submitted to a β-radiation counting (LS 6500—Beckman, Beckmann Instruments Inc., Fullerton, CA, USA).

#### Immunofluorescence for GFAP and GLT-1

For immunofluorescence studies, after the separation of astrocytes by agitation, the cells were transferred to 12-well sterile culture plates (TPP®) containing glass coverslips pretreated with poly-L-lysine (5 mg/50 mL, Sigma). Once confluent, astrocyte cells were then fixed with methanol for 3 min. Coverslips containing the fixed cells were incubated for 1 h with 10% Normal Horse Serum (Vector Laboratories), followed by primary antibodies to astrocyte specific markers; rabbit anti-glial fibrillary acidic protein (anti-GFAP, 1:500, Sigma-Aldrich #G9269) and mouse anti-SLC 1A2 [GLT-1 subtype (mouse 1:500, Sigma-Aldrich #WH0006506M10)] or HACCs were incubated with rabbit anti-GFAP in combination with primary antibody to neurons specific marker; mouse anti-NeuN (1:750, Millipore #MAB 377) antibodies, overnight. After incubation, the coverslips were rinsed in 0.01 M PBS and incubated with Cy3-conjugated donkey anti-mouse and FITC-conjugated donkey anti-rabbit secondary antibody (1:250, Jackson Immunoresearch) for 2 h, washed and to finish the cells were incubated with DAPI (1 μg/ml Thermo Scientific #28718-90-3) for 5 min. After washes, cells were fixed with mounting medium (Fluromount) and visualized under fluorescence light. Immunofluorescence to GFAP, GLT-1, and NeuN were qualitatively performed to assess astrocyte cells primary cultures homogenity.

### Stereotaxia and central microinjections

The rats were anesthetized with ketamine (75 mg/Kg body weight, i.p.) and xylazine (10 mg/Kg body weight, i.p.) using a stereotaxic apparatus (David-Kopf, Tujunga, CA, EUA). Unilateral stain- less steel guide cannulas (12-mm-long-cannula- 22 gauge Small Parts, Miami Lakes, FL, USA) were implanted in the left lateral cerebral ventricle, according to brain atlas of Paxinos and Watson (1997), as follows; −0.5 mm rostral to the interaural line, left +1.4 mm from the medial suture and −3.9 mm deep from the skull. The cannula was fixed in the skull using dental acrylic resin and two metal screws. A tight-fitting mandril was kept inside the guide cannula to avoid occlusion. After surgery, the rats received a prophylactic injection of penicillin and streptomycin (0.1 mL/100 g 1.200.000 IU, Fort Dodge, Campinas, SP, Brazil). The drugs were microinjected icv into the lateral ventricle using a Hamilton microsyringe (Hamilton, Reno, NV, USA) connected by a PE-10 polyethylene tubing to a dental needle. At the time of the experiment, the mandril was carefully removed and the dental needle was inserted into the guide cannula. The final volume microinjected was 0.5 μL.

### Catheterization

One day before the cardiorespiratory experiment and 5–6 days after stereotaxic surgery, the rats were anesthetized with ketamine and xylazine (75 and 10 mg/Kg, respectively, i.p). A polyethylene catheter (PE-10 connected to PE-50; Clay Adams, Parsippany, NJ, USA) was inserted into the abdominal aorta through the femoral artery for cardiovascular recording. The catheters were tunneled subcutaneously and exteriorized in the back of the neck. After catheterization surgery, animals were housed in individual cages.

### Cardiovascular and respiratory recordings in awake rats

On the day of the experiment (24 h after artery catheterization), the rats were placed in Plexiglas chambers (5 l) for respiratory measurements. The femoral artery catheter was then flushed with heparinized saline to prevent clotting and connected to a pressure transducer (BRPL2, WPI, Sarasota, FL, USA) coupled to an amplifier and an acquisition system (PowerLab, ADInstruments, Bella Vista, NSW, Australia) running LabChart 5.0 (ADInstruments, Bella Vista, NSW, Australia). MAP (mmHg) and HR (bpm) were derived from the pulsatile arterial pressure (PAP, mmHg). Baseline BP and HR were recorded for 60 min. After establishing baseline cardiovascular parameters, ventilatory activity was measured using the whole-body plethysmographic method according to (Malan, 1973). After each icv microinjection, the plexiglas chamber was closed and air flow was suspended for short periods (3 min). Before breath recordings, we performed a volume calibration via injecting 1 ml of air into the chamber and subsequently captured air displacement by the respiratory cycles via a pressure differential transducer connected to a signal amplifier (ML141 Spirometer, 196 PowerLab; ADInstruments). Tidal volume (V_T_, mL) and minute volume (V_M_, mL) were derived from the breath according to the method reported by Bartlett and Tenney (1970).

### Animals and *in vivo* experimental protocols

Male wistar rats (*R. norvegicus*) weighing 260–280 g (total 64 animals) were obtained from the Biotechnology Center of Federal University of Paraiba and from the School of Medicine of Ribeirao Preto, University of São Paulo, Brazil. The animals were maintained in individual cages at controlled room temperature (21 ± 2°C), 12-h light-dark cycle (lights were on from 6:00 a.m. to 6:00 p.m.) and with tap water and food (Labina®, Purina, SP, Brazil) *ad libitum*.

#### Experimental protocols

All studies were performed in awake and freely moving rats. The animals were assigned to one of three experimental protocols: neuroendocrine, drinking, and cardiorespiratory responses. In the neuroendocrine and drinking protocols the rats were divided in four groups, according to treatment; (1) saline (0.15 M NaCl); (2) ANG-II (0.1 M); (3) FCt (50 mM) and (4) FCt (50 mM) + ANG-II (0.1 M). The neuroendocrine protocol was designed to measure AVP and OT plasma release. Five-to-six days after stereotaxic surgery and 24 h before the experiment the rats were placed in the experimental room. On the experimental day, microinjections were performed in 5 min intervals. Five minutes after the second icv microinjection, the animals were decapitated and trunk blood was collected for radioimmunoassay analyses. The drinking protocol was designed to measure cumulative water and sodium (1.5% NaCl) intake. Two days after stereotaxic surgery, and 72 h before the experiments, the animals were placed in an individual metabolic cages provided with water and sodium solution (1.5% NaCl) burettes. During the experiment, food was removed, and cumulative water and sodium intake were measured at 5, 15, 30, 60, 120, 240 min after the last microinjection. In the cardiorespiratory protocol, 5 days after stereotaxic surgery the animals were anesthetized for femoral artery catheterization. The cardiorespiratory protocol was designed to measure mean arterial pressure (MAP, mmHg), heart rate (HR, bpm), respiratory frequency (fR, beats/min), V_T_ (mL), and V_M_ (mL). Twenty-four hours after catheterization, the animals were placed in a plethysmographic chamber to record ventilatory parameters and the femoral artery catheter was connected to a pressure transducer to record cardiovascular parameters. Before beginning the cardiorespiratory recording, at least 60 min period was allowed for stabilization of parameters and animal adaptation. We performed icv microinjection of ANG-II (0.1 M) before (as control) and 5, 10, 15, and 30 min after icv saline (0.15 M NaCl) or FCt (50 mM) pretreatment.

### ANG-II and fluorocitrate (FCt) dilution

ANG-II (Millipore #CAS 447-91-3) was dissolved in sterile saline (0.15 M) at a final concentration of 0.1 M (Reis et al., 2010). Fluorocitrate (Sigma-Aldrich #F9634) solutions were prepared according to a protocol based on the study by Costa et al. (2013) and Paulsen et al. (1987). In brief, 2 mg of barium fluorocitrate was dissolved in 10 μL of HCl 1 M until it formed a homogeneous solution and Na_2_SO_4_ 0.1 mM was added dropwise in order to precipitate Ba^++^ ions. Next, 20 μl of Na_2_HPO_4_ 0.1 M was added and the solution was centrifuged at 3,000G for 10 min. The supernatant was isolated and diluted in saline solution (0.15 M NaCl) to a final concentration of 50 mM. The pH of the final FCt solution was adjusted to 7.4 with sodium bicarbonate (Reagen, Rio de Janeiro, Brazil).

The toxic effect of FCt seems to depend not only for the dose but also on the volume and central area microinjected (Paulsen et al., 1987, 1988a,b; Hassel et al., 1992). Studies by Paulsen et al. (1987) suggested that 1 nmol/1 μL of FCt microinjected intrastriatally is not accompanied by seizure and acts as a selective, reversible glial cell inhibitor. On the other hand, studies by Mirsattari et al. (2008) observed that FCt 0.84 nmol/0.125 μL microinjected intracortically, induces seizure. Additionally, intratriatal microinjection of FCt, at the same dose (1 nmol) as Paulsen et al. (1987) but in a larger volume (i.e., 1 μL), induce seizures (Hassel et al., 1992). In our study the FCt dose (50 mM/500 nL) was chosen by considering the drug dilution in the total estimated (400–550 μL) cerebrospinal fluid volume (i.e., adult rats ~300 g; Frankmann, 1986; Pegg et al., 2010). It is important to note that in our study icv microinjection of FCt was not accompanied by seizures or death over 4 h of observation (i.e., drinking behavioral protocol) and the physiological changes induced by this glial cell inhibitor were reversible a few minutes after microinjection, as we observed in the cardiorespiratory protocol.

### Determination of plasma hormone levels

After decapitation, trunk blood was collected into cold plastic tubes containing heparin (10 μL per mL of blood) to measure the plasma OT and AVP levels. Plasma was obtained after centrifugation at 3,000 rpm for 25 min at 4°C and stored at −70°C until specific extraction and immunoassay procedures were performed. AVP and OT were extracted from 1 ml of plasma with acetone and petroleum ether. All measurements were performed in duplicate. The inter- and intra-assay variations were 12.6 and 7% for OT, and 17.5 and 3.3% for AVP, respectively. AVP and OT plasma measurements were performed using specific radioimmunoassay techniques, as previously described (Haanwinckel et al., 1995; Elias et al., 1997).

### Histological procedure

Histological examination of the microinjection was performed after completion of all experiments. The rats were anesthetized via ip injection of ketamine and xylazine (75 and 10 mg/Kg, respectively), and 0.5 μl of filtered Evan's Blue 2% (Vetec, Química Fina Ltda, Rio de Janeiro, RJ, Brazil) dye was microinjected in the lateral ventricle as a marker for the microinjection site. Animals were transcardially perfused with 10% formaldehyde and the brains were removed and kept in sucrose (30%) solution for 24 h. Sections (60 μm) from hypothalamus were cut on a cryostat (Leica, Wetzlar, Germany). Histological sections were analyzed using Paxinos and Watson (1997) atlas as reference and considered positive when guide cannula tracing were visualized in bright field microscopy.

## Results

### Effects of ANGII on hypothalamic astrocyte culture cells-induced glutamate uptake

**Figure 1:**
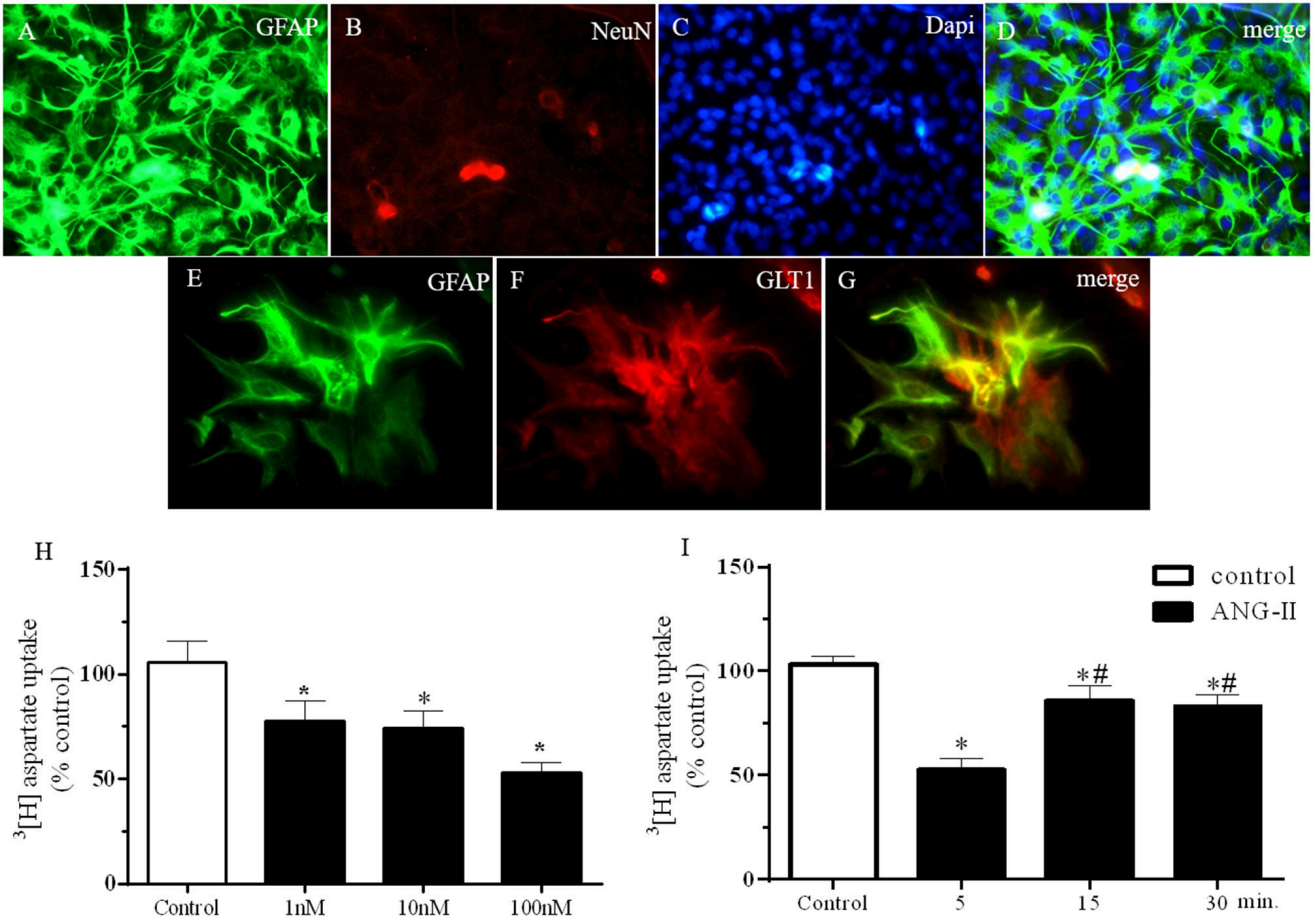
ANG-II decrease ^3^[H]-Aspartate uptake in hypothalamic astrocyte primary cultures cells. **(A–G)** Immunofluorescence showing GFAP **(A,E)**, NeuN **(B)**, DAPi **(C)**, and GLT1 **(F)** in primary astrocyte culture. Panel **(G)** merge of panels **(A–C)**. **(H)** ^3^[H] Aspartate uptake after ANG-II stimulation at 1, 10, or 100 nM during 5 min. **(I)** ^3^[H] Aspartate uptake after ANG-II stimulation at 100 nM during 5 (*n* = 11), 15 (*n* = 18), or 30 (*n* = 26) min. Differences among groups were calculated by One-Way ANOVA followed by the Newman-Keuls post test. Values were expressed as % of control. *p < 0.05 vs. control; ^#^*p*< 0.05 vs. 15 and 30 min.

As represented by Figures 1A–G, HACs primary culture homogeneity were qualitatively determined, as showed in the colocalization between GFAP and DAPi (Figure 1D) and GFAP and GLT-1 (Figure 1G) and confirmed by no immunoreactivity for NeuN (Figure 1B). HACc were treated for 5 min with ANG-II at 1, 10, and 100 nM as shown in Figure 1H. ANG-II stimulation induced a decrease in the ^3^H-aspartate uptake in primary hypothalamic astrocytes [*F*_(3, 37)_ = 6.7, *P* = 0.001]; similar results were obtained 5, 15, and 30 min after ANG-II stimulation at 100 nM [*F*_(3, 105)_ = 10.4, *P* < 0.0001]. It is important to note that 5 min (53 ± 5% control) ANG-II stimulation was more efficient in reducing ^3^H-aspartate uptake than 10 (86 ± 7.5% control) or 15 min (83 ± 5.6% control; Figure 1I).

### Effects of FCt pretreatment on central ANG-II-induced AVP and OT secretion

**Figure 2:**
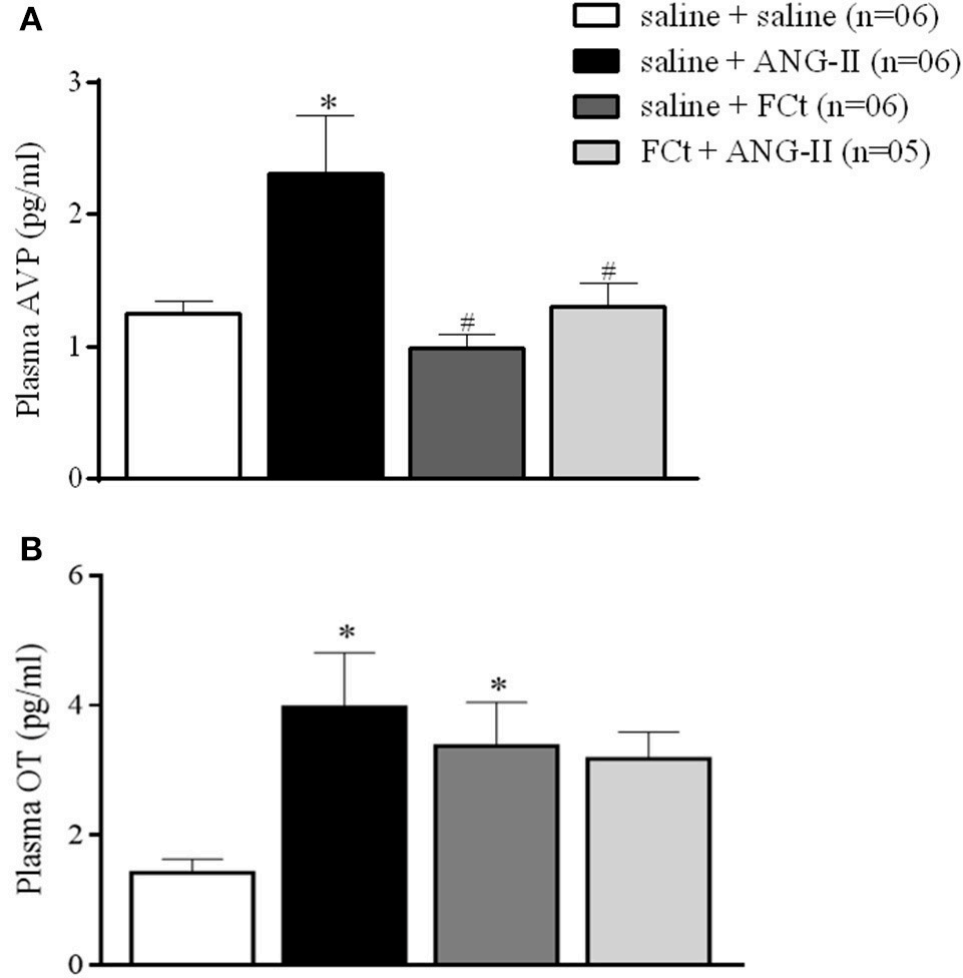
Effect of icv saline, ANG-II or FCt microinjection on vasopressin [AVP **(A)**] and Oxytocin [OT **(B)**] plasma release after local saline or FCt pretreatment in awake rats. Differences among groups were calculated by One-Way ANOVA followed by the Newman–Keuls post test. Values were expressed as means ± SEM. *p < 0.05 vs. control group (saline + saline). ^#^*p* < 0.05 vs. saline + ANG-II group.

Figure 2 shows plasma concentration of AVP (Figure 2A) and OT (Figure 2B) induced by icv saline or ANG-II in animals pretreated with saline or FCt. For AVP secretion (Figure 2A), we found a statistically significant effect of treatment [*F*_(3, 19)_ = 5.7, *p* = 0.006]. As expected, central ANG-II induced an increase in AVP secretion [2.3 ± 0.4 vs. 1.3 ± 0.1 pg/mL]. Microinjection of FCt alone did not change AVP plasma levels (1 ± 0.1 vs. 1.3 ± 0.1 pg/mL), however FCt pretreatment attenuated ANG-II-induced AVP plasma increase (1.3 ± 0.2 vs 2.3 ± 0.4 pg/mL). For OT secretion (Figure 2B) we again found a significant effect of treatment [*F*_(3, 18)_ = 3.4, *p* = 0.04]. Central ANG-II induced an increase in OT plasma levels [4.0 ± 0.8 vs. 1.4 ± 0.2 pg/mL]. In addition, FCt alone increased OT plasma levels (3.4 ± 0.7 vs. 1.4 ± 0.2 pg/mL), however it did not change the ANG-II-induced effect on OT release (3.2 ± 0.4 vs. 4.0 ± 0.8 pg/mL).

### Effects of FCt pretreatment on central ANG-II-induced water and sodium intake

**Figure 3:**
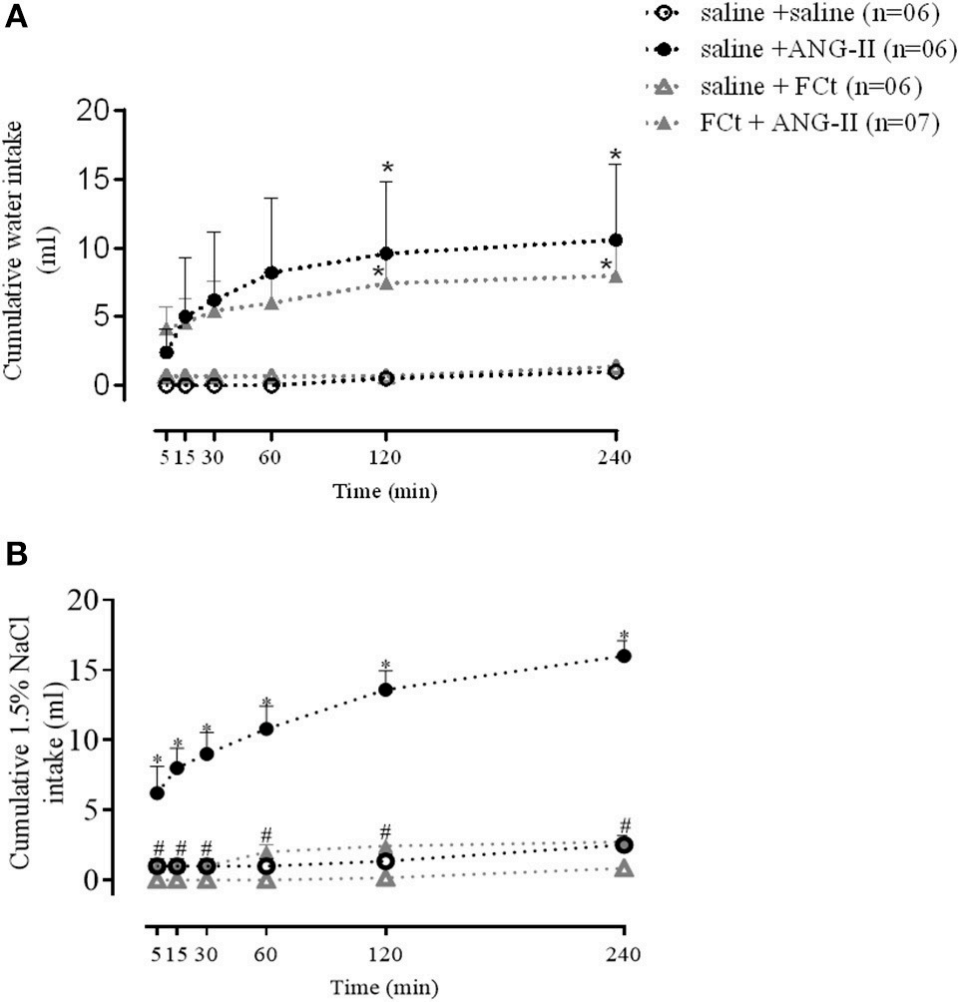
Effect of icv saline, ANG-II or FCt microinjections on water **(A)** and sodium [1.5% NaCl **(B)** intake] 5, 15, 30, 60, 120, and 240 min after saline or FCt pretreatment in awake rats. Differences among groups were calculated by Two-Way ANOVA followed by the Newman–Keuls post test. Values were expressed as means ± SEM. *p < 0.05 vs. control group (saline + saline). ^#^*p* < 0.05 vs. saline + ANG-II group.

The results summarized in Figure 3 show cumulative water and sodium intakes induced by central ANG-II (icv) in saline or FCt pretreated animals before and 5, 15, 30, 60, 120, and 240 min after ANG-II injection. Data analysis indicated a significant increase in the water intake as function of treatment [*F*_(3, 120)_ = 4.3, *P* = 0.0001], but this parameter did not change as a function of time [*F*_(5, 120)_ = 1.2, *P* = 1] or interaction between factors [*F*_(5, 120)_ = 0.3, *P* = 1]. As indicated in Figure 3A, central ANG-II significantly increased cumulative water intake (7 ± 1 vs. 0.25 ± 0.2 mL/4 h), being this effect was not changed by previous FCt administration (7 ± 1 vs. 6 ± 0.6 mL/4 h). The results also showed a significant increase in sodium intake as function of treatment [*F*_(3, 120)_ = 240, *P* < 0.0001] and time [*F*_(5, 120)_ = 13, *P* < 0.0001], as well as an interaction between factors [*F*_(15, 120)_ = 4.4 *P* < 0.0001]. Furthermore, central ANG-II-induced a marked increase in sodium intake (16 ± 1 vs. 2.5 ± 0.7 mL/4 h), which was blocked by previous FCt microinjection (2.7 ± 0.3 vs. 16 ± 1 ml/4 h) (Figure 3B). Nevertheless, FCt alone did not change cumulative water (0.25 ± 0.2 vs. 0.7 ± 0.1 mL/4 h; Figure 3A) or sodium intake (0.8 ± 0.3 vs. 2.5 ± 0.7 mL/4 h; Figure 3B).

### Effects of FCt pretreatment on central ANG II-induced cardiorespiratory responses

**Figure 4:**
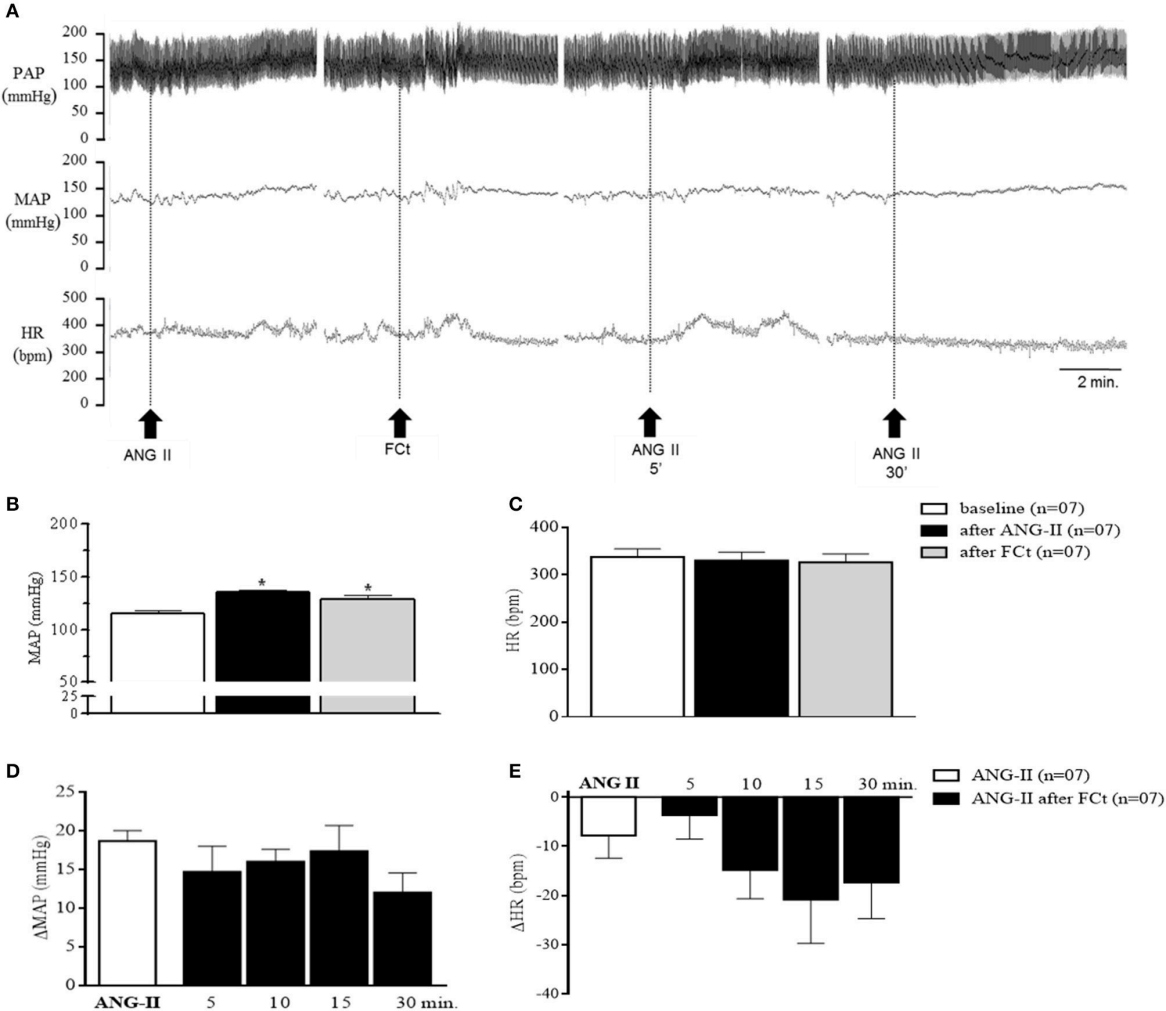
Effect of icv ANG-II or FCt microinjection on baseline cardiovascular parameters. **(A)** Representative tracings of one rat showing changes in pulsatile arterial pressure (PAP, mmHg), mean arterial pressure (MAP, mmHg), and baseline heart rate (HR, bpm) induced by icv ANG-II before and 5 and 30 min after FCt pretreatment. Arrows indicate icv ANG-II or FCt microinjections. **(B,C)** Changes in baseline MAP and **(B)** heart rate **(C)** before and after icv microinjection of ANG-II or FCt. **(D,E)** changes in MAP **(D)** and HR **(E)** to icv microinjection of ANG-II before and 5, 10, 15, and 30 min after local FCt pretreatment in awake rats. Differences among groups were calculated by One-Way ANOVA followed by the Newman–Keuls post test. Values were expressed as means ± SEM. *p < 0.05 vs. control.

The representative tracings in Figure 4A show changes in PAP, MAP, and HR in response to central ANG-II microinjection in awake rats in the presence or absence of FCt. One-way ANOVA analysis revealed a significant difference in baseline MAP as a function of treatment [*F*_(2, 18)_ = 11.5, *p* = 0.0006]. Microinjection of ANG-II (135 ± 2.8 vs. 116 ± 2 mmHg) or FCt (129 ± 3.6 vs. 116 ± 2 mmHg) increased baseline MAP, but no significant changes were observed in baseline HR (Figures 4A–C). Results showed that FCt pretreatment did not alter the central ANG-II-induced pressor response [*F*_(4, 25)_ = 1, *p* = 0.4; Figures 4A,D] or baseline HR [*F*_(4, 25)_ = 1.2, *p* = 0.35; Figure 4E]. As observed in Table 1, saline (0.15 M) microinjection did not change baseline PAP, MAP, or HR, neither cardiovascular changes were induced by central ANG-II.

**Table 1:** Changes in baseline MAP (Δ, mmHg) and HR (Δ, bpm) induced by icv **ANG-II** microinjections before (control) and 5, 10, 15, and 30 min.

**Saline**	**Δ MAP (mmHg)**	**Δ HR (bpm)**
Control	26.1 ± 3.6	−32.5 ± 20.3
5′	23.4 ± 3.9	−40.4 ± 20.9
10′	22.2 ± 5.3	−23.5 ± 17.7
15′	11.3 ± 6.5	−9.6 ± 5.3
30′	30.9 ± 8.3	−57.7 ± 34.7

*After icv **saline** pretreatment. Values are expressed as means ± SEM and were obtained in 3–5 rats/saline + ANG-II group*.

**Figure 5:**
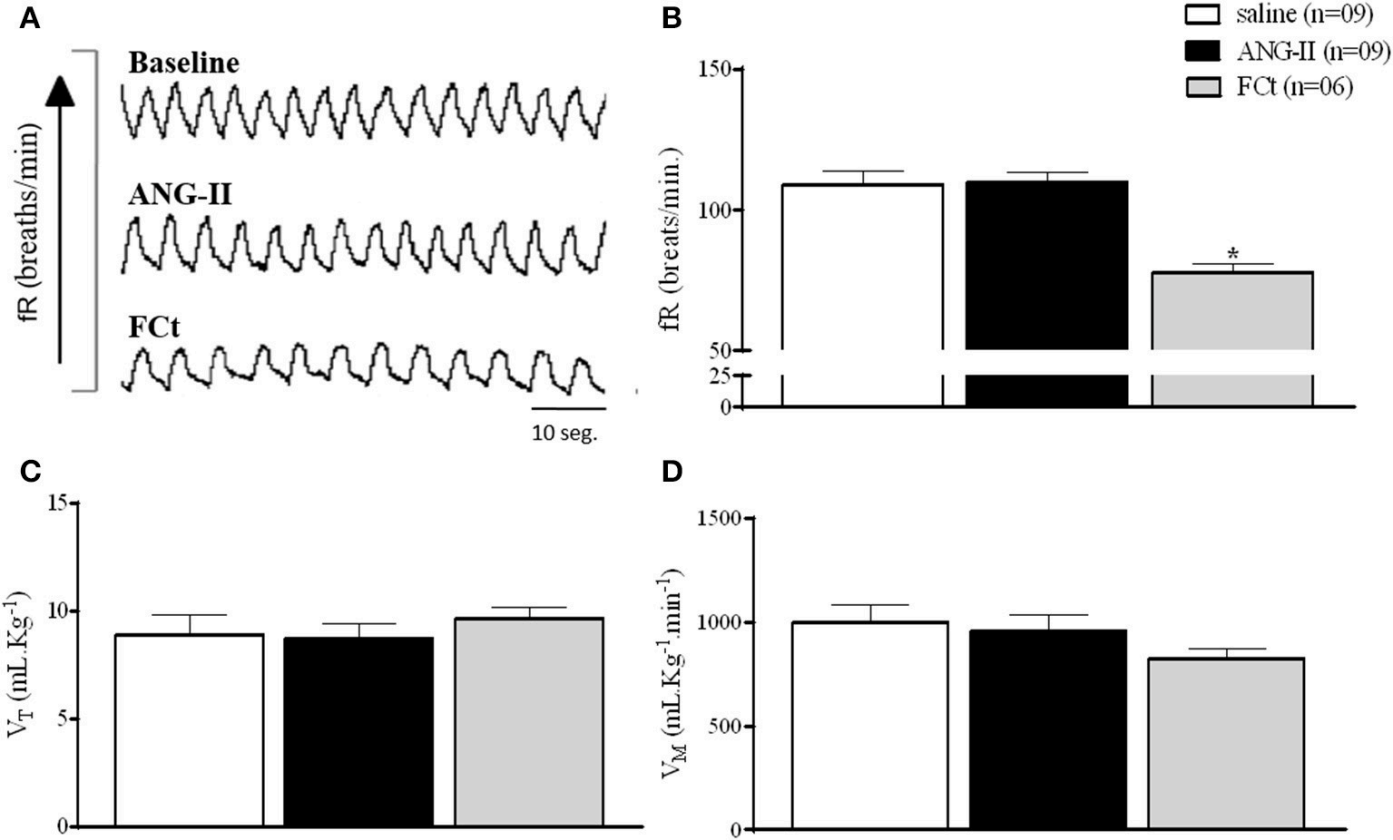
Effect of icv ANG-II or FCt microinjection on baseline respiratory parameters. **(A)** Representative tracings of one rat showing the baseline and changes in the fR (breaths/min) induced by icv microinjection of ANG-II and FCt. Arrows indicate icv ANG-II or FCt microinjections. **(B–D)** Respiratory frequency [fR, breaths/min **(B)**]; Tidal Volume [V_T_ mL.Kg^−1^ **(C)**] and Volume Minute [V_M_ mL.Kg-1.min^−1^ **(D)**] after microinjection icv of saline, ANG-II, or FCt. One-Way ANOVA followed by the Newman–Keuls post test. Values were expressed as means ± SEM. *p < 0.05 vs. saline.

Figure 5A show typical respiratory recordings of one representative rat. Central ANG-II did not change baseline fR, V_T_, or V_M_ (Figures 5B–D and Table 2). Interestingly, central FCt microinjection decreased breathing at rest [77.7 ± 2.9 vs. 109. ± 4.6 breaths/min; *F*_(2, 22)_ = 13, *P* = 0.0002; Figures 5A,B], but did not change V_T_ [9.7 ± 0.5 vs. 8.9 ± 0.9 mL.Kg^−1^; *F*_(2, 22)_ = 0.35, *P* = 0.7; Figure 5C] or V_M_ [827.2 ± 47 vs. 1002 ± 83 mL.Kg^−11^ (*F*_(2, 22)_ = 1.2, *P* = 0.3) (Figure 5D). In addition, icv microinjection of saline + ANG-II or FCt + ANG-II did not change respiratory fR, V_T_, or V_M_ at rest (Table 2).

**Table 2:** Changes in baseline fR, VT, and VM induced by icv **ANG-II** microinjections before (control) and 5, 10, 15, and 30 min.

**Saline**	**fR (breats/min.)**	**VT (mL.Kg^−1^)**	**VM (mL.Kg^−1^.min^−1^)**
**SALINE**			
Control	109.8 ± 5.5	9 ± 2.1	978.5 ± 184.3
5′	107.9 ± 5.1	8.9 ± 0.9	832.9 ± 196.1
10′	106.7 ± 8.2	6.9 ± 1.5	767.3 ± 212.3
15′	112.5 ± 0.3	7.4 ± 1.1	832 ± 126
30′	104.8 ± 7.7	6.9 ± 0.6	742.4 ± 128.9
**FCt**			
Control	109.6 ± 5.9	8.6 ± 0.7	950.7 ± 98.2
5′	95.6 ± 6.7	9.9 ± 0.7	933.2 ± 59.7
10′	102.3 ± 4.7	10.2 ± 0.5	1044 ± 50.2
15′	98.8 ± 8.7	9.8 ± 0.7	960.2 ± 92.8
30′	92 ± 6.2	9.7 ± 0.8	870.5 ± 31.1

*After icv **saline or FCt** pretreatment. Values are expressed as means ± SEM and were obtained in 3–5 rats/saline + ANG-II group and 7–9 rats/ANG-II + FCt group*.

### Histology

**Figure 6:**
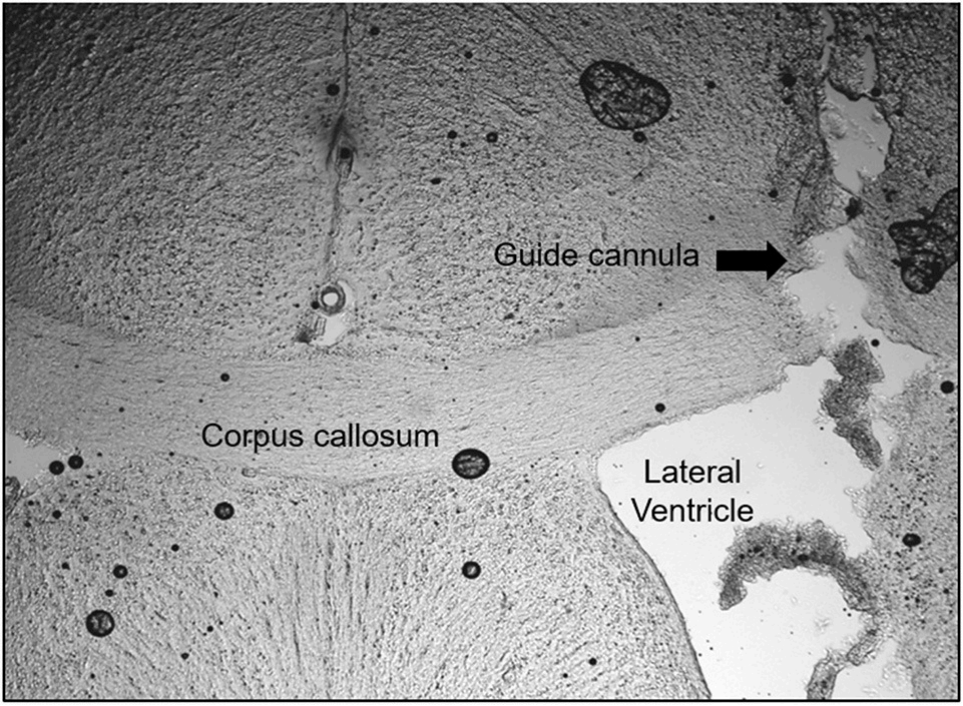
Sites of the unilateral microinjections into the lateral ventricle. Photomicrograph of a coronal section (60 μm) of the brain of one rat, representative of the group, showing microinjections tracing in the lateral ventricle. Arrows show the tract of the cannula.

Figure 6 shows a photomicrograph of a coronal brain section illustrating the unilateral track of the microinjection site in the left lateral ventricle of one representative animal. For all experimental protocols, only rats with positive histological verification of the cannula site were included in the analyses.

## Discussion

The present set of *in vitro* data suggest that ANG-II stimulates hypothalamic astrocytes, decreasing extracellular glutamate clearance. Furthermore, *in vivo* studies showed that central acute FCt-mediated glial inhibition increased OT secretion; decreased salt intake and interestingly increased pressure and decreased respiratory frequency at rest. In addition, our data showed that central acute inhibition of glial cells decreased AVP secretion and salt intake induced by central ANG-II. Taken together, our results suggest that CVOs glial cells are important in integrating signaling which acts to modulate body fluid and cardiorespiratory homeostasis, including central effects induced by central ANG-II. A summary of our results are illustrated in the Graphical Abstract.

### ANG II effects in the hypothalamic astrocyte culture cells

Our *in vitro* findings indicated that ANG II decreased glutamate uptake in HACc, as indirectly assessed in the present study by [^3^H]-aspartate transport across the membrane. Studies by Boudaba et al. (2003) observed that dehydration increased SON postsynaptic excitatory currents (mEPSCs), which is mediated by a diminished astrocyte glutamate uptake. In addition, recent studies by Stern et al. (2016) showed that ANG II decreased PVN astrocyte glutamate currents. Taken together, these studies suggest that dehydration and ANG II decrease astrocyte glutamate transport, inducing an increase in the extracellular glutamate bioavailability, which in turn, contributes to an increase in neuronal activity (see Graphical Abstract).

### Role of circumventricular glial cells in the neuroendocrine responses induced by central ANG-II

The present data showed that FCt alone did not change AVP secretion. However, FCt pretreatment decreased central ANG-II-induced AVP release, suggesting that CVOs glial cells modulate AVP release driven by central ANG-II. This mechanism could be mediated by an ANG-II-induced decrease in astrocyte glutamate transport, leading to an increased glutamatergic neurotransmission, as suggested by Stern et al. (2016) and supported by our *in vitro* studies. Thus, here we suggest, as illustrated in the Graphical Abstract, that ANG-II induced AVP secretion is modulated by astrocyte-mediated extracellular glutamate clearance, which is impaired by FCt, an inhibitor of glial cell metabolism, leading to a decreased participation of these cells in glutamine-glutamate cycle (Paulsen et al., 1987, 1988a,b; Hassel et al., 1992). Next, we reported that central FCt microinjection induced alone a significant increase in OT secretion, suggesting that baseline activity of oxytocinergic neurons is under CVOs glial cells modulation. In agreement, studies by Di et al. (2013) showed that FCt promotes a significant reduction in post-synaptic inhibitory currents evoked in SON magnocellular neurons. Furthermore, it is known that OT neurons, under basal conditions, are inhibited by GABAergic inputs (Haam et al., 2012; Morton et al., 2014), whose post-synaptic activity is modulated by surrounding astrocytes (Potapenko et al., 2013). In light of exciting evidence, we hypothesized that under basal conditions, CVOs glial cells act to inhibit activity of hypothalamic oxytocinergic neurons. A number of studies have suggested that acute stimuli elucidate different, opposing mechanisms to modulate magnocellular vasopressinergic and oxytocinergic neurons (Haam et al., 2012; Di et al., 2013; Morton et al., 2014; Stachniak et al., 2014). Accordingly, central acute FCt attenuated ANG-II induced AVP secretion, as discussed above, but did not change ANG-II induced OT secretion, suggesting CVOs glial cells are important to modulate ANG-II-induced AVP secretion, but are not critical to modulate ANG-II-induced OT secretion.

### Role of circumventricular glial cells in the behavioral responses induced by central ANG-II

The present study provides additional evidence that central ANG-II promotes a significant increase in water and sodium intake in awake rats (Fitzsimons, 1980; Beresford and Fitzsimons, 1992; Saad et al., 2004; Reis et al., 2007, 2010; Matsuda et al., 2016). ANG-II receptors are strongly expressed in CVOs (Lenkei et al., 1997; Gebke et al., 1998), especially in the SFO, the main site involved in the control of sodium and water intake.

Despite the abundance of glial cells processes surrounding SFO neurons (Watanabe et al., 2006), the relationship between these glial cells in modulating drinking behavior is currently poorly understood. Our results suggest that CVOs glial cells are not involved in modulating water intake, but seem to be involved in ANG-II-induced sodium intake. Supported by our *in vitro* findings, and accordingly to the report of Stern et al. (2016), we suggest that central ANG-II-induced sodium intake could be modulated by astrocyte-mediated extracellular glutamate clearance (see Graphical Abstract). Another explanation was postulated by Noda's group; in a set of elegant studies, they showed that SFO glial cells, through Na^+^ sensitive channels (Na_x_), are involved in sodium intake (Watanabe et al., 2006; Shimizu et al., 2007; Matsuda et al., 2016). However, it appears that CVOs glial cells are associated with an inhibitory mechanism, which acts to modulate sodium intake, since elevated extracellular Na+ concentration increased firing of GABAergic interneurons in the SFO of wild-type, but not of Na_x_-Knockout mice (Shimizu et al., 2007; Matsuda et al., 2016). Additionally, in a different study, the same group showed that lactate released by sodium-sensitive astrocytes (Na_x_-positive) increased local GABAergic interneuron activity, which seems to be involved in the inhibitory modulatory pathway driving sodium intake in dehydrated mice (Watanabe et al., 2006; Shimizu et al., 2007; Matsuda et al., 2016). As previously described, Zielke et al. (2007) reported that FCt microdialysis into the hippocampus of freely moving rats considerably increased interstitial lactate levels. In that regard, we also hypothesize that sodium intake induced by central ANG-II could be impaired by FCt-induced increased interstitial lactate. However, interaction of ANG-II, lactate and CVOs glial cells in modulating drinking behavior needs to be further investigated.

### Role of circumventricular glial cells in the cardiorespiratory responses induced by ANG-II

Our study provides additional evidence that central ANG-II induces the pressor responses in awake rats, as previously described (Saad et al., 2004; Reis et al., 2010). In addition, pressor response induced by central ANG-II seems to be modulated by SFO angiotensinergic neurons projecting to pre-sympathetic PVN neurons (Bains et al., 1992; Bains and Ferguson, 1994). Bains et al. (1992) observed that PVN AT1_R_ antagonism attenuated a pressor response induced by SFO electrical stimulation. In addition, our results show that CVOs glial inhibition induces a pressor response in awake rats. In that regard, blockade of PVN astrocyte glutamate transporter increased renal sympathetic outflow and induced a pressor response in anesthetized rats (Bardgett et al., 2014; Stern et al., 2016). Furthermore, our results showed that central FCt did not change the ANG-II induced pressor response in awake rats, suggesting that CVOs glial cells are not critical to modulate central ANG-II induced pressor response. On the other hand, studies by Stern et al. (2016), focused on PVN pre-sympathetic neurons, indicated that ANG-II-induced sympathoexcitation and pressor responses are mediated by astrocyte-neuron interactions in the PVN. Thus, although our studies have shown that CVOs glial cells are not critical to modulate central ANG-II pressor response, those cells appear to play a critical role in mediating cardiovascular homeostasis.

Next, we observed that central ANG-II did not promote significant changes in respiratory frequency rate, tidal volume or ventilation in freely-moving awake rats. Studies by Zubcevic et al. (2013) have shown that icv ANG-II infusion (for 7 days) changed the pattern of phrenic nerve activity during the post-inspiration phase (PI or stage I expiration), typical of sympathetic-respiratory coupling, demonstrating that chronic activation of angiotensinergic receptors in CVOs cells seems to be involved in the sympathetic-respiratory coupling which promotes changes in the respiratory pattern. It is important to note that chronic ANG-II activation in these hypothalamic regions increases the expression of AT1_R_, thereby increasing plasma ANG-II sensitivity (Wei et al., 2009). Concerning that, central ANG-II-induced ventilatory changes could be observed in pathological conditions. Supporting our finding, studies by Walker and Jennings (1998) did not observe changes in ventilation promoted by intravenous ANG-II in awake rats. Considering that ANG-II appears to exert ventilatory effects via different mechanisms, our results suggest that central ANG-II is not involved in baseline respiratory outflow in awake rats. Our data showed that FCt-inhibited CVOs glial cells attenuate respiratory frequency at rest, which could be mediated by a decreased glutamine-glutamate cycle (Paulsen et al., 1987, 1988a,b; Hassel et al., 1992). Indeed, many other studies have suggested that brainstem astrocytes are involved in the respiratory network (Gourine et al., 2005; Erlichman et al., 2010; Sobrinho et al., 2017). Our findings provide new evidence supporting that CVOs glial cells are involved in the maintenance of breathing control at rest, in awake rats. However, the interaction between CVOs glial cells and the respiratory brainstem nucleus remains to be fully evaluated.

In summary, our *in vitro* findings showed that ANG-II decreased glutamate uptake in HACc. In addition, our *in vivo* findings suggest that CVOs glial cells modulate OT secretion, blood pressure and breathing at rest. Furthermore, CVOs glial cells are important in mediating ANG-II induced salt intake behavior and AVP secretion, which we propose is modulated by ANG-II inhibited glutamate uptake by surrounding CVOs astrocytes.

## Author contributions

AF, JC: designed experiments; AF, JC, SR: performed and analyzed experiments; AF, JC: wrote the manuscript; JdBA, CB, MF-S, LE, SR, JA-R, and VB: review the manuscript.

### Conflict of interest statement

The authors declare that the research was conducted in the absence of any commercial or financial relationships that could be construed as a potential conflict of interest.

## Abbreviations

ANG-II: Angiotensin II
HACc: Astrocyte culture cells
CVOs: Circumventricular organs
EAAT: Excitatory amino acid transporter
FCt: Fluorocitrate
GLT1: Glial-specific glutamate transporters
GLAST: Glutamate aspartate transporter
HR: Heart rate
icv: Intracerebroventricular
MAP: Mean arterial pressure
V_M_: Minute Volume
OVLT: Organum vasculosum of the lamina terminalis
OT: Oxytocin
PVN: Paraventricular nuclei
PAP: Pulsatile arterial pressure
fR: Respiratory frequency
SFO: Subfornical organ
V_T_: Tidal volume
AVP: Vasopressin.
